# The Differential Effects of Immunosuppressants on Hepatitis E Virus Replication and the Triggered Inflammatory Responses in Macrophages

**DOI:** 10.1111/jvh.70118

**Published:** 2025-12-16

**Authors:** Jiahua Zhou, Kuan Liu, Patrick P. C. Boor, Qiuwei Pan, Ibrahim Ayada

**Affiliations:** ^1^ Department of Gastroenterology and Hepatology Erasmus MC‐University Medical Center Rotterdam the Netherlands; ^2^ Department of Surgery Erasmus MC Transplant Institute, University Medical Center Rotterdam Rotterdam the Netherlands

**Keywords:** hepatitis E virus, immunosuppressants, inflammatory responses, macrophages, NLRP3 inflammasome

## Abstract

Organ transplant recipients are at high risk of developing chronic infection when exposed to hepatitis E virus (HEV), which can rapidly progress to liver fibrosis and cirrhosis. Macrophages play a key role in the response to the infection and disease progression. However, the interactions amongst immunosuppressants, macrophages, the course of HEV infection and activation of inflammatory response remain unclear. In this study, we generated M0, M1 and M2 macrophages from the human THP‐1 cell line. These macrophages were then infected with HEV and treated with different immunosuppressants. We visualised viral infection using laser confocal microscopy, and quantitatively analysed viral replication and inflammatory responses by bulk sequencing, RT‐qPCR, ELISA and Western blotting. We found that the M1 inflammatory macrophages exhibited the highest, while M2 macrophages had the lowest levels of viral RNA. Genome‐wide transcriptome analysis indicated that viral, inflammation and immunity‐related pathways were predominantly upregulated by HEV infection. Dexamethasone exerted potent inhibitory effects on inflammatory response in macrophages. Mycophenolic acid (MPA) demonstrated inhibitory effects on viral replication, IL‐1β and TNF‐α expression, whereas mTOR inhibitors had the opposite effects, and tacrolimus showed no clear effect. In conclusion, immunosuppressants can differentially affect HEV replication and the subsequent inflammatory responses in macrophages.

## 1 Introduction

Hepatitis E virus (HEV) is a single‐stranded positive‐sense RNA virus that belongs to the Hepeviridae family and the Orthohepevirus genus. In humans, HEV infection is primarily caused by Orthohepevirus A, which comprises eight genotypes. Humans are most susceptible to genotype (GT) 1, 2, 3 and 4 [1]. HEV causes sporadic cases and endemic outbreaks, leading to approximately 20 million infections with roughly 70,000 deaths annually worldwide [2].

Chronic HEV infection is defined as the persistence of HEV RNA in blood or stool for more than 3 months. The primary routes of chronic infection include (1) blood transfusion: receiving blood or blood products from HEV‐positive donors, (2) organ transplantation: receiving organs from HEV‐positive donors and (3) environmental exposure: consuming HEV‐contaminated water or undercooked pork. In a meta‐analysis, the pooled estimated prevalence of HEV infection amongst solid organ transplant patients was found to be 20.2%, based on a total of 4557 individuals, and anti‐HEV IgM seroprevalence is 1.5% [3]. Organ transplant recipients require long‐term immunosuppressive therapy, which weakens the immune system and increases the risk of HEV infection and chronicity. In Europe, immunosuppressed organ transplant recipients, exposed to GT3 or GT4 HEV, face a high risk of developing chronic hepatitis, which may rapidly progress to liver fibrosis and cirrhosis [4]. Currently, the main treatment strategies for chronic HEV infection include reducing immunosuppression and antiviral therapy.

The substantial risk of developing chronic HEV infection in organ recipients is associated with the use of immunosuppressive medications [5]. These agents are widely administered after organ transplantation and include pharmacological agents or small molecule agents which act by inhibiting cytokine release (such as calcineurin inhibitors and corticosteroids) or by inhibiting the cell cycle (including anti‐metabolites and mTOR inhibitors). These pharmacological drugs are specifically designed to modulate immune responses to prevent graft rejection [6]. HEV infection caused liver diseases are intricately related to dysregulation of immune responses [7]. Inflammatory responses serve as the first line of defence in the immune responses and have gained significant attention, particularly regarding the role of the NLRP3 inflammasome [8]. The NLRP3 inflammasome is essential for regulating inflammatory responses, and its activation can lead to the release of inflammatory factors such as mature IL‐1β [9], resulting in liver inflammation. Furthermore, the interferon response to diverse viral infections universally induces hundreds of interferon‐stimulated genes (ISGs) [10], thereby creating a broad‐spectrum intracellular antiviral state.

Our previous studies have demonstrated that HEV can infect and replicate in human macrophages, which activates the NLRP3 inflammasome [11]. Activation of the NLRP3 inflammasome can trigger the release of inflammatory factors such as mature IL‐1β, further aggravating the inflammatory responses in the liver. Therefore, understanding how different immunosuppressants influence viral replication and infection‐related inflammatory responses in the context of HEV infection is critical. In this study, we aim to investigate how immunosuppressants affect HEV infection and the triggered inflammatory response in macrophages.

## 2 Materials and Methods

### 2.1 Ethics Statement

The study is based on cell lines and does not involve any direct intervention with human participants. Therefore, in accordance with the regulations of the relevant ethics committee, ethical approval is not required for this research.

### 2.2 Reagents

Prednisone (Pre), dexamethasone (Dex), rapamycin (Rapa), everolimus (Ever), tacrolimus (Tac), cyclosporine (CSA) and mycophenolic acid (MPA) were purchased from MedChemExpress (The Netherlands) and dissolved in the optimal stock concentrations.

### 2.3 Macrophage Culture and Differentiation

Human monocytic THP‐1 cells were cultured in RPMI 1640 medium supplemented with 30 ng/mL of phorbol 12‐myristate 13‐acetate (PMA, Sigma‐Aldrich Chemie BV) at 37°C for 48 h to generate M0 macrophages. Following PBS washing, M0 macrophages were treated with 100 ng/mL LPS (Lipopolysaccharides from *Escherichia coli* O55:B5, Merck Life Science NV) and 25 ng/mL IFN‐γ (Recombinant Human IFN‐γ, Immunotools) for M1 macrophages polarisation or 25 ng/mL IL‐4 (Miltenyi biotec) and IL‐13 (Miltenyi biotec) for M2 macrophages polarisation for an additional 2 days. The morphology of THP‐1 macrophages was assessed by transmission microscopy (EVOS FL Cell Imaging System, ThermoFisher). The expression of monocyte and macrophage markers was analysed using flow cytometry. Flow cytometric analysis was conducted on a FACS SymphonyTM A3 (BD Biosciences), and the resulting data was analysed using FlowJo software (version 10.10.0; Tree Star Inc.). Cells were harvested and labelled for commonly used monocyte/macrophage markers CD14 (301,841, Biolegend), CD32 (12‐0329‐42, ThermoFisher), HLA‐DR (17‐9956‐42, ThermoFisher), CD80 (IM1853U, Beckman), DC‐SIGN (25‐2099‐42, ThermoFisher) and CD163 (12‐1639‐41, ThermoFisher) to assess their phenotypes. Cells were first gated based on their size and granularity (FSC vs. SSC) as well as viability, using 7‐AAD(00–6993‐50, ThermoFisher). Markers expression were plotted in histograms, with gates set based on isotype controls.

### 2.4 HEV Cell Model and Virus Production

Genotype 3 HEV models were established using a plasmid construct containing the full‐length HEV genome (genotype 3 Kernow‐C1 p6 clone, GenBank accession number JQ679013) [12] and produced in human liver Huh7 cells as previously described [13]. Briefly, viral RNA was synthesised in vitro using the mMESSAGE mMACHINE T7 Transcription Kit (Life Technologies Europe BV). Subsequently, Huh7 cells were electroporated with p6 full‐length HEV RNA to generate infectious models. Huh7 cells harbouring the infectious genotype 3 HEV were seeded into T175 flasks. HEV particles were harvested by repeated freezing and thawing for 2 times, and filtered by 0.2 μm filters.

For the re‐infection model, infectious THP‐1‐p6 cells were first generated by electroporating THP‐1 cells with full‐length p6 HEV RNA. These cells were then subjected to PMA‐induced differentiation over 48 h in T175 flasks to derive M0‐p6 macrophages. The supernatant containing HEV particles secreted by M0‐p6 cells was harvested and clarified using a 0.2 μm filter.

### 2.5 Virus Inoculation and Immunosuppessants Treatment

Prior to infection, macrophages were washed twice with PBS to remove the residual reagents following differentiation. Subsequently, macrophages were inoculated with HEV particles (2.3 × 10^7^ copy numbers/mL) from Huh7‐p6, and cultured for 24, 48, or 72 h.

Prior to infection, Huh7 cells were washed twice with PBS to remove the residuals. Subsequently, cells were inoculated with HEV particles (1.4 × 10^5^ copy numbers/mL) from M0‐p6, and cultured for 24, 48, or 72 h.

For treatment, the macrophages were washed as above. Next, macrophages were inoculated with HEV particles and treated with immunosuppressants for 48 h.

### 2.6 Immunofluorescence Staining and Confocal Imaging

Macrophages and Huh7 cells were inoculated with HEV for 72 h. Cells were fixed by exposure to 4% (w/v) paraformaldehyde (PFA) for 10 min at room temperature. Subsequently, the cells underwent three washes with PBS, each lasting 5 min. Permeabilization was achieved by treating the cells with PBS containing 0.2% (vol/vol) Triton X‐100 for 5 min, followed by two additional PBS washes lasting 5 min each. Next, the cells were subjected to a blocking step using 1% bovine serum albumin (BSA) in PBS at room temperature for 1 h. Then anti‐dsRNA antibody (1:500, mouse mAb; Scicons J2) was diluted in 1% BSA incubated with cells at 4°C overnight. Afterwards, cells were incubated with secondary antibody at room temperature for 1 h. Nuclei were counterstained with DAPI (4,6‐diamidino‐2‐phenylindole; Invitrogen). Finally, fluorescence imaging was performed using a Leica SP5 confocal microscope with a 20× objective to analyse the stained cellular structures. Double stranded RNA (dsRNA) of HEV (red) and the nucleus marker DAPI (blue) were examined under confocal microscopy.

### 2.7 Quantification of Viral Replication and Inflammatory Genes

Viral RNA and inflammatory genes were quantified by SYBR‐Green‐based real‐time PCR (RT‐qPCR) with the Applied Biosystems SYBR Green PCR Master Mix (Thermo Fisher Scientific Life Sciences). GAPDH served as a housekeeping gene for normalising gene expression, employing the 2^−(ΔΔCt)^ method.

### 2.8 Genome‐Wide RNA Sequencing and Data Analysis

M0 macrophages were inoculated with HEV particles for 24 h. In parallel, non‐infected M0 macrophages were cultured under the same conditions to serve as negative controls. For RNA extraction, total RNA was isolated using the Macherey‐Nagel NucleoSpin RNA II Kit (Bioke, Netherlands). The extracted RNA was initially quantified by using the Bioanalyzer RNA 6000 Picochip. RNA sequencing was performed by Novogene, employing a paired‐end 150 bp (PE 150) sequencing strategy.

Principal Component Analysis (PCA) of the whole transcriptome was conducted to compare samples based on all gene expression levels. The principal component coefficients and heatmap of inflammatory genes and ISGs between groups were generated using R language (R‐4.3.0 version). The interactions amongst multiple genes may be involved in viral and immune pathways. KEGG (Kyoto Encyclopaedia of Genes and Genomes) pathway analysis was conducted based on differentially expressed genes, with KEGG pathways showing an adjusted *p*‐value(padj) < 0.05 considered significantly enriched. RNA sequencing data are publicly available at Data Archiving and Networked Services (DANS) https://doi.org/10.17026/LS/W57FUZ.

### 2.9 Western Blot

Cells were harvested with DTT and protein buffer (1:9), and proteins in supernatant were collected using the methanol precipitation method. All proteins were heated at 95°C for 5 min, then loaded onto a 10% and 15% sodium dodecyl sulphate polyacrylamide gel (SDS‐PAGE). The gels were separated at 90 V for 120 min and electrophoretically transferred onto a polyvinylidene difluoride (PVDF) membrane (pore size: 0.45 mm; Thermo Fisher Scientific Life Sciences) for 120 min at an electric current of 250 mA. After transfer, the membrane was blocked with blocking buffer (Li‐Cor Biosciences) and then incubated overnight at 4°C with primary antibodies: rabbit anti‐NLRP3 (1:1000), anti‐NFkB p65 (1:1000), anti‐Pro‐IL‐1β (1:1000), anti‐Cleaved IL‐1β (1:1000), or mouse anti‐β‐actin (1:1000). Following three time washes, the membrane was incubated for 1 h at room temperature with anti‐rabbit or anti‐mouse IRDye‐conjugated secondary antibodies (1:5000; Li‐Cor Biosciences). After additional washing, protein bands were detected using Odyssey 3.0 Infrared Imaging System.

### 2.10 Elisa

The concentrations of IL‐1β and TNF‐α were measured using enzyme‐linked immunosorbent assay (ELISA) with the Human IL‐1 beta/IL‐1F2 Quantikine ELISA Kit (Bio‐Techne, Netherlands) and the Human TNF alpha Uncoated ELISA Kit (Thermo Fisher Scientific Life Sciences).

### 2.11 MTT Assay

10% Thiazolyl Blue(MTT, Sigma‐Aldrich Chemie BV) was added to the cells seeded in 48‐well plates, and the cells were maintained at 37°C with 5% CO2 for 4 h. The medium was then removed, and 100 μL of DMSO was added to each well. The absorbance of each well was measured using a microplate absorbance reader (BIO‐RAD) at a wavelength of 570 nm.

### 2.12 Statistics

Statistical analysis was conducted using a non‐paired, non‐parametric test (Mann–Whitney U test; GraphPad Prism 8.0.2). All results were presented as mean ± SD (*p* < 0.05*; *p* < 0.01**)

## 3 Results

### 3.1 HEV Infects naïve and Polarised Macrophages

**FIGURE 1:**
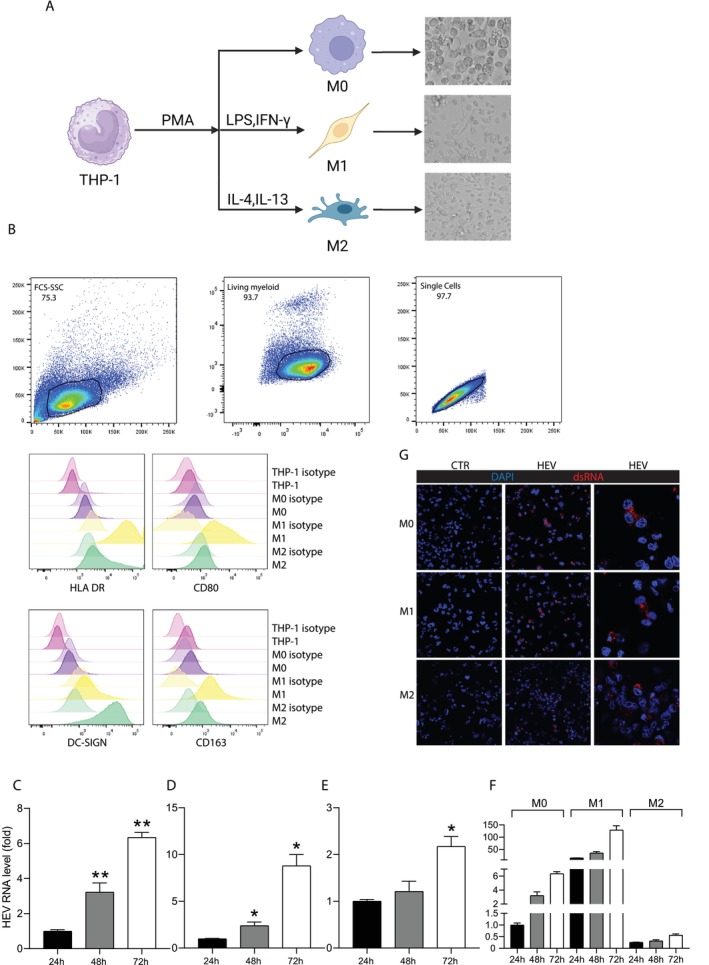
Characterising HEV infection in three types of human macrophages. (A) Schematic overview of polarising THP‐1 macrophages and the morphology of macrophages. The schematic illustration was generated in Biorender. (B) Gating strategies for flow cytometry analysis of macrophage markers and flow cytometry‐based phenotyping of THP‐1 monocyte‐derived macrophages. (C–F) Quantitative RT‐PCR analysis of HEV viral level in macrophages inoculated with HEV and cultured for 24, 48, or 72 h (*n* = 4–5). (G) Macrophages were inoculated with HEV for 72 h. Viral dsRNA (red) and nucleus marker DAPI (blue) were examined under confocal microscopy. *p < 0.05; **p < 0.01.

Human monocytic THP‐1 cells can be polarised into naïve M0 macrophages, and M0 macrophages can be further polarised into M1 pro‐inflammatory or M2 anti‐inflammatory subtypes. Following well‐established protocols [14], we generated these three types of macrophages (M0, M1 and M2) and confirmed their phenotypes based on morphology (Figure 1A). Additionally, we verified their surface CD markers to further validate their subtypes (Figure 1B and Figure S1A,B).

Next, we inoculated M0, M1 and M2 macrophages with cell culture‐derived genotype 3 HEV infectious particles. Quantification of HEV genome using RT‐qPCR revealed a significant increase in viral replication from day 1 to day 3 post‐inoculation (Figure 1C–E). Of note, M1 macrophages appeared to have the highest, whereas M2 macrophages had the lowest levels of viral RNA (Figure 1F). Immunofluorescent staining analysis revealed HEV double‐stranded RNA (dsRNA), the replicating intermediate, in infected macrophages (Figure 1G).

To investigate whether macrophages can produce infectious HEV particles, cell culture supernatant was collected from macrophages electroporated with the full‐length HEV genome. Subsequently, Huh7 cells were inoculated with the collected supernatant, and inoculation of Huh7 cells produced HEV was served as a positive control (Figure S2A). Quantification of the HEV RNA by RT‐qPCR and immunofluorescence analysis of HEV dsRNA confirmed that macrophages produce HEV particles that are infectious and capable of replication in Huh7 cells (Figure S2B,C). Collectively, these results demonstrated that human macrophages can support HEV infection and replication and produce infectious HEV particles.

### 3.2 HEV Infection Triggers Inflammatory Response in Macrophages

**FIGURE 2:**
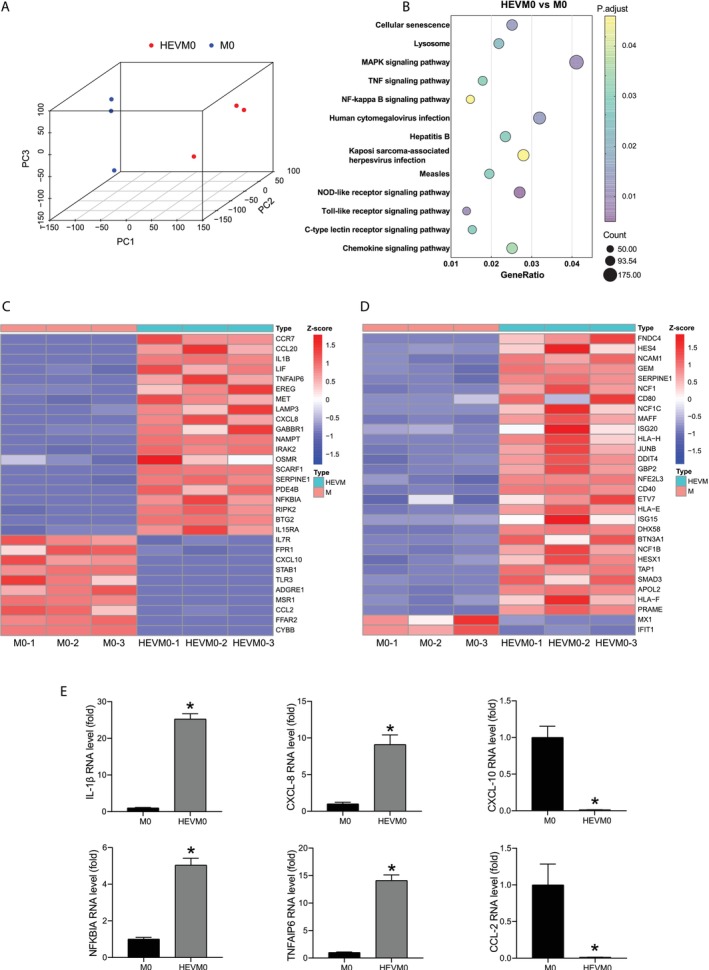
Responses to HEV infection in M0 macrophages. (A) PCA plots based on genome‐wide transcriptome of M0 macrophages inoculated with HEV for 24 h. (B) Viral and immune pathway enrichment from the genome‐wide transcriptomic data. (C) Cluster analysis of top 30 genes of ‘HALLMARK_INFLAMMATORY_RESPONSE’ from transcriptome (D) Cluster analysis of 30 genes of interferon‐stimulated genes (ISGs) from transcriptome. (E) Validation of 6 top inflammation‐associated genes in M0 macrophages inoculated with HEV for 24 h by RT‐qPCR (*n* = 4). *p < 0.05.

To better understand the responses of macrophages to HEV infection, genome‐wide transcriptomic analysis was conducted on M0 macrophages 24 h after inoculation. Principal component analysis (PCA) clearly distinguished uninfected from infected macrophages (Figure 2A). Pathway analysis revealed that viral and immune response related pathways were predominately upregulated by HEV infection amongst the top 36 significant pathways. According to the KEGG pathway catalogue [15], these included 8 pathways ranging from ‘Human cytomegalovirus infection’ to ‘Measles’ and ‘NOD‐like receptor signalling pathway’ to ‘Chemokine signalling pathway’, illustrating primary effects of HEV infection on M0 macrophages (Figure 2B). The remaining five pathways are also closely related to immunity and inflammation triggered by pathogens. The ‘Cellular senescence’ pathway involves the release of inflammatory cytokines that exert autocrine, paracrine and endocrine activities [16]. The ‘Lysosome’ pathway serves as a safeguard, preventing pathogens from reaching the cytoplasm before being degraded [17]. The ‘MAPK signalling pathway’ has a broad range of effects on viral infection and inflammatory responses [18]. The ‘TNF signalling pathway’ plays a critical role in various physiological and pathological processes, including the modulation of immune responses and the induction of inflammation [19]. Lastly, the ‘NF‐kappa B signalling pathway’ is implicated in the pathogenesis of chronic inflammatory diseases [20].

‘HALLMARK_INFLAMMATORY_RESPONSE’ is a gene set, defining inflammatory response in Gene Set Enrichment Analysis (GSEA) [21]. The heatmap exhibited the top 30 genes of relative gene expression from the ‘HALLMARK_INFLAMMATORY_RESPONSE’ gene set in the whole transcriptome, comprising 20 inflammatory genes and 10 anti‐inflammatory genes (Figure 2C). Similarly, an additional heatmap illustrated the 30 significantly differentially expressed ISGs [10] (Figure 2D). Subsequently, we verified the expression of top‐regulated genes using RT‐qPCR, confirming that HEV triggered a robust activation of inflammatory gene expression, including IL‐1β, CXCL‐8, NFKBIA and TNFAIP6, while leading to the down‐regulation of anti‐inflammatory gene expression such as CXCL‐10 and CCL‐2 (Figure 2E).

### 3.3 The Effects of Different Immunosuppressants on HEV Replication in Macrophages

Given that organ transplant patients infected with HEV bear an extremely high risk of developing chronic hepatitis because of immunosuppressive medications [22], we comprehensively profiled the effects of clinically used immunosuppressants on HEV replication in macrophages. As a pilot test, we initially used a relatively low concentration (1 μM) of seven types of immunosuppressants to treat HEV infected M0 macrophages and observed varying effects on viral replication (Figure S1C).

**FIGURE 3:**
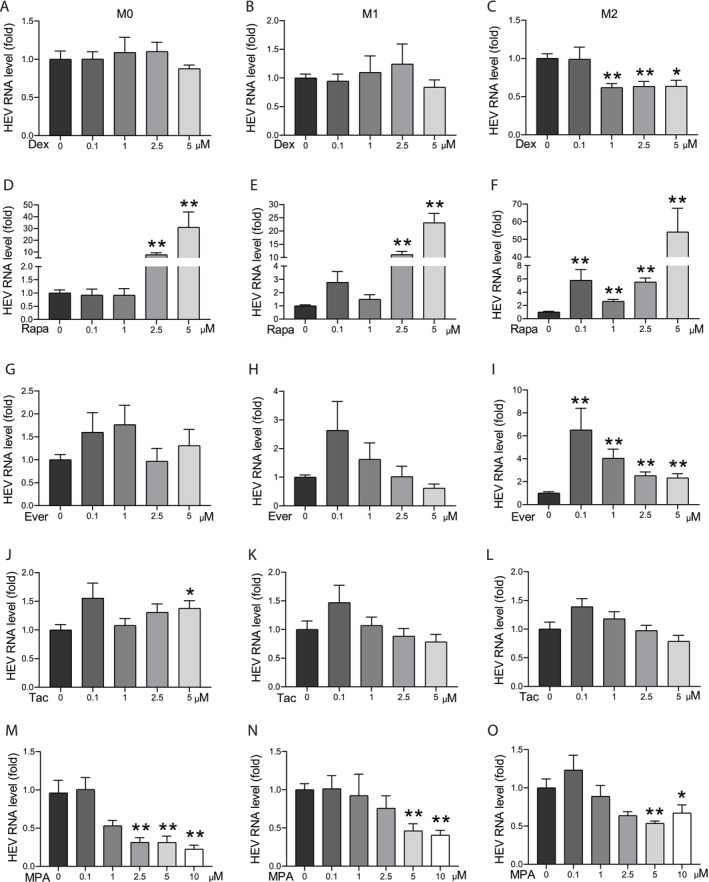
Immunosuppessants on HEV infection in macrophages. (A–O) Quantitative RT‐PCR analysis of HEV RNA level in macrophages after HEV infection and treatment by immunosuppressants for 48 h (*n* = 6). *p < 0.05; **p < 0.01.

Next, we focused on the widely used immunosuppressants: the steroid dexamethasone, two mTOR inhibitors (rapamycin and everolimus), tacrolimus and mycophenolic acid (MPA). We evaluated their dose‐dependent effects in M0, M1 and M2 macrophages. Dexamethasone assists in induction and maintenance of immunosuppression and reverse acute cellular rejection in transplantation [23]. Dexamethasone had a minimal effect on HEV replication in macrophages, but only high concentrations slightly inhibited viral replication in M2 macrophages (Figure 3A–C). mTOR inhibitors serve as calcineurin inhibitor sparing agents in organ recipients with kidney dysfunction, hepatocellular carcinoma, or de novo neoplasms [23]. Rapamycin dose‐dependently increased viral replication in all three types of macrophages, and effects were prominent at concentrations of 2.5 and 5 μM (Figure 3D–F). For example, 5 μM of rapamycin increased HEV viral RNA levels by 31, 23 and 54‐fold in M0, M1 and M2 macrophages, respectively (Figure 3D–F), although this concentration had significant inhibitory effects on cell viability (Figure S1F). Regarding everolimus, pro‐viral effects were only significant in M2 macrophages, with a reversed dose‐dependent trend (Figure 3G–I). Tacrolimus did not affect viral replication and macrophage viability (Figure 3J–L and Figure S1F). MPA is typically administered as an immunosuppressive regimen when signs of acute allograft rejection are observed in transplant patients [23], and it exhibited a significant inhibition on HEV replication in all three types of macrophages in a dose‐dependent manner (Figure 3M–O). Thus, different immunosuppressants exert differential effects on HEV replication in macrophages.

### 3.4 The Effects of Immunosuppressants on HEV‐Triggered Inflammatory Responses in Macrophages

Macrophages are a key immune cell type that mediate virus‐triggered pathological inflammation. Inflammasome activation, in particular the NLRP3 inflammasome, has been implicated in the HEV‐triggered inflammatory response in macrophages [11, 24]. IL‐1β is a hallmark of NLRP3 inflammasome activation in macrophages [8].

**FIGURE 4:**
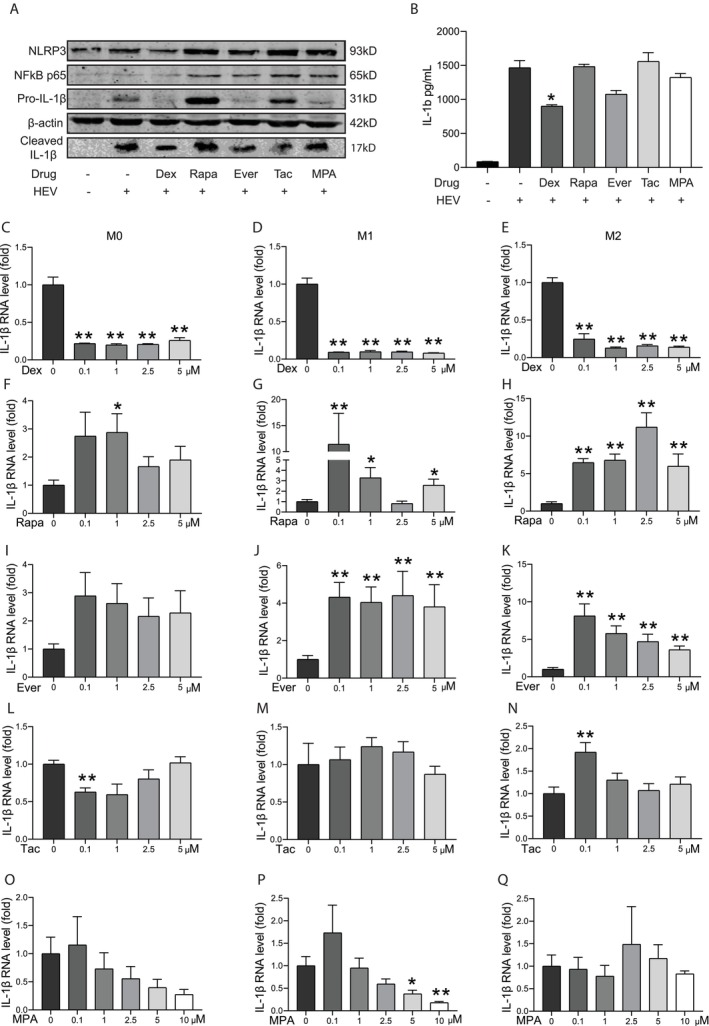
Immunosuppressants on HEV‐triggered inflammatory response in macrophages. (A) Mature IL‐1β in supernatant or pro‐IL‐1β, NF‐kB p65 and NLRP3 in lysates were determined by western blot in M0 macrophages after HEV infection and treatment by immunosuppressants (1 μM) for 48 h. (B) IL‐1β protein level was quantified by ELISA in M0 macrophage after HEV infection and treatment by immunosuppressants (1 μM) for 48 h (*n* = 4). (C–Q) Quantitative RT‐PCR analysis of IL‐1β mRNA level in M0, M1 and M2 macrophages after HEV infection and treatment by immunosuppressants for 48 h (*n* = 6). *p < 0.05; **p < 0.01.

We first primarily assessed the effects of seven immunosuppressive agents (1 uM) on IL‐1β gene expression in HEV‐infected M0 macrophages. Prednisone, dexamethasone, and tacrolimus appear to inhibit, while rapamycin and everolimus increase IL‐1β expression (Figure S1D). At protein levels detected by western blotting (Figure 4A) or ELISA (Figure 4B), HEV infection dramatically triggered mature IL‐1β release, while the inhibition was most prominent by dexamethasone treatment. Moderate inhibition of TNF‐α was observed by dexamethasone, everolimus, and MPA, while rapamycin significantly increased TNF‐α production (Figure S1E).

Finally, we investigated the effects of dexamethasone, rapamycin, everolimus, tacrolimus and MPA on IL‐1β gene expression with a series of concentrations in M0, M1 and M2 macrophages. Dexamethasone consistently and potently inhibited IL‐1β expression in all three macrophages (Figure 4C–E). In contrast, rapamycin and everolimus stimulated IL‐1β expression (Figure 4F–K). Tacrolimus had no major effects (Figure 4L–N), while MPA had inhibitory effects (Figure 4O–Q). Collectively, although different immunosuppressants exerted differential effects, steroids had the most potent inhibitory effects on HEV‐triggered inflammatory responses in macrophages.

## 4 Discussion

Organ transplant patients routinely take immunosuppressants to prevent organ rejection. Our previous study has demonstrated that different immunosuppressive regimens can differentially affect the course of HEV infection in human liver cells [25, 26]. Furthermore, we also found that HEV can infect human macrophages and activate inflammasomes via the NF‐κB/NLRP3 pathway to drive the inflammatory response [11, 24]. Building on our previous research, this study further demonstrates that different immunosuppressive regimens can differentially affect the course of HEV infection and the activation of the inflammatory response in human macrophages.

Macrophages play a key role in inflammasome‐mediated defence against infections in the liver [27]. We and others have previously shown that both human macrophages are permissive to HEV infection [28], which triggers robust activation of the NLRP3 inflammasome [11, 24]. In this study, we used the human monocytic leukaemia THP‐1 cell line model, which is frequently used as a surrogate for primary human macrophages. Upon exposure to stimuli, THP‐1 cells can differentiate into macrophage‐like cells that closely resemble primary human macrophages and can also be polarised into inflammatory M1 and anti‐inflammatory M2 macrophages through standardised differentiation processes [14]. In our experiments, we found that dexamethasone and MPA exhibited inhibitory effects on viral replication and IL‐1β expression in macrophages, while mTOR inhibitors had the opposite effects, and tacrolimus showed no clear effects. The concentrations of these immunosuppressants used in this study generally reflect achievable blood concentrations in patients [25]. Amongst these drugs, we particularly focused on the effects of dexamethasone and MPA.

In clinical practice, dexamethasone has been utilised since the early years of organ transplantation, and it has also been widely used to treat a variety of other diseases, including autoimmune diseases and COVID‐19. The use of dexamethasone has been shown to reduce the pathogen clearance rate of SARS‐CoV‐2 and stimulate viral replication [29]. However, dexamethasone does not increase susceptibility to HEV infection in cell model [25] and animal models [30]. Moreover, when treating COVID‐19 patients with dexamethasone, there is a significant reduction in the inflammatory cytokine storm, which is generated by a range of immune cells, including macrophages, and includes inflammatory factors such as IL‐1β and TNF‐α [31]. In our results, dexamethasone exhibited only a slight inhibitory effect on viral replication in M2 macrophages at higher concentrations but significantly inhibited IL‐1β and TNF‐α expression in macrophages. Therefore, the powerful anti‐inflammatory effects of dexamethasone seem indisputable; however, its impact on HEV replication in immune cells remains to be debated, especially in the context of the limited related research available.

MPA is the active metabolite of mycophenolate mofetil and mycophenolate sodium. It blocks inosine‐5′‐monophosphate dehydrogenase, thereby suppressing T‐cell proliferation. Due to its lack of renal toxicity, MPA is often used in combination to de‐escalate or discontinue calcineurin inhibitors [23]. Our previous study demonstrated that MPA inhibits HEV replication in human liver cell models [25]. Additionally, in renal transplant patients, reducing the dosage of MPA did not effectively halt the continued viral replication of HEV, which persisted despite receiving ribavirin treatment [32]. In this study, we demonstrated that MPA dose‐dependently inhibited HEV replication in macrophages, and also exerted an inhibitory effect on IL‐1β and TNF‐α expression in macrophages. Despite the limited research on this topic, loading MPA into naïve macrophage‐secreted exosomes significantly enhances its anti‐inflammatory and antioxidant effects in vitro compared to free drugs, aiding in the rescue of cardiac myoblasts following inflammatory injury [33]. Therefore, the effect of MPA on inflammation may vary depending on the microenvironment.

Of note, there are some limitations in this study. First, chronic hepatitis E in organ transplant patients is primarily caused by GT3 and GT4 HEV. We only have a GT3 model available, and therefore future research of using a GT4 HEV model is needed for validation. Second, beyond the THP‐1 cell model, it would be interesting to use primary macrophages derived from human liver tissue for further validation. Finally, our results are based on in vitro models and a single drug, and thus validation in vivo and drug combination are important before translating into clinical implications. Furthermore, in this study, we produced HEV stocks by cell culture supernatant with cell lysate of infected Huh7 cells. It is known that supernatant mainly contains enveloped HEV (eHEV) particles released via natural secretion, whereas the lysate primarily contains non‐enveloped, naked virions accumulated intracellularly, along with some eHEV [34]. Thus, our viral preparation consisted of a mixture of both naked and enveloped HEV particles, which are known to utilise different cellular entry mechanisms [34]. The use of such a combined supernatant‐lysate preparation is well‐established in in vitro studies [12, 35], as it maximises viral yield and provides a robust system for achieving efficient infection. Future research should investigate whether macrophages are permissible to both forms of HEV.

In conclusion, this study comprehensively profiled the clinically used immunosuppressants on HEV replication and inflammatory response in human macrophages. While these immunosuppressants exert differential effects, dexamethasone appears to have the most potent anti‐inflammatory effect. Although our findings cannot directly refine or dictate the optimal immunosuppressive regimen for organ transplant patients infected with or at risk of HEV infection, they hold significant importance for advancing and achieving this goal.

## Author Contributions

Jiahua Zhou performed the experiments and analysed data. Jiahua Zhou wrote the draft of the manuscript. Kuan Liu and Patrick P.C. Boor performed some experiments. Ibrahim Ayada and Qiuwei Pan conceived the ideas, designed and discussed experiments, supervised the project, and extensively edited the manuscript.

## Conflicts of Interest

The authors declare no conflicts of interest.

## Supporting information


**Figure S1:** jvh70118‐sup‐0001‐Figures.pdf.
**Figure S2:** jvh70118‐sup‐0001‐Figures.pdf.

## Data Availability

The data that support the findings of this study are available from the corresponding author upon reasonable request.
