# Supplementary material for: The Differential Effects of Immunosuppressants on Hepatitis E Virus Replication and the Triggered Inflammatory Responses in Macrophages
Source: J Viral Hepat. 2025 Dec 16;33(1):e70118. doi: 10.1111/jvh.70118 (PMC12706704; doi:10.1111/jvh.70118)
Supplement: Supplementary file 1 — Figure S1: jvh70118‐sup‐0001‐Figures.pdf. Figure S2: jvh70118‐sup‐0001‐Figures.pdf. [file JVH-33-0-s001.pdf]

Supplementary Figure 1

Supplementary Fig. 1

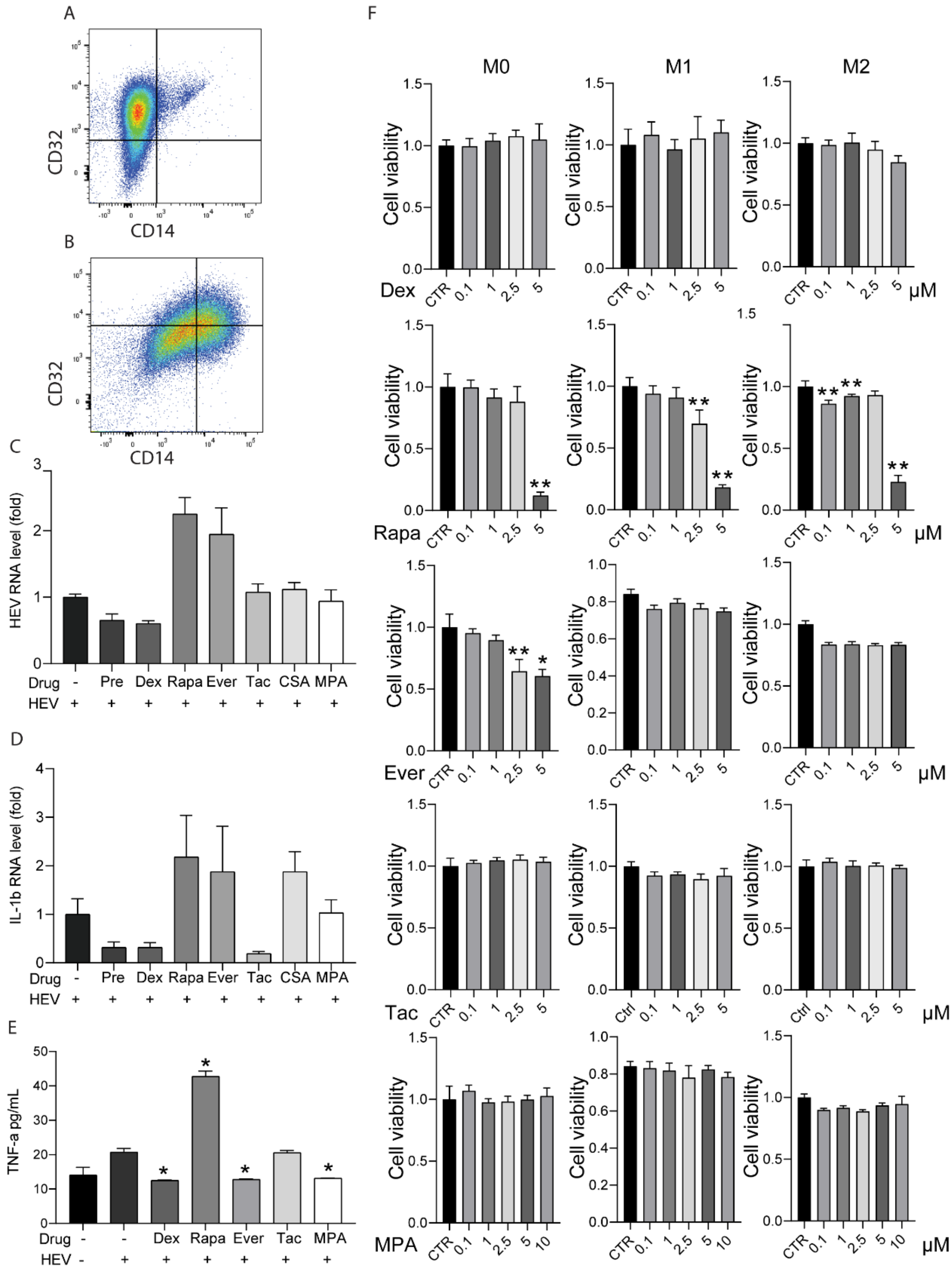

A,B, Characterization of THP-1 monocytes and M0 macrophages by measuring the expression of CD14 and CD32

C,D, Quantitative RT-PCR analysis of HEV and IL-1β mRNA level in M0 macrophages after HEV infection and treatment by immunosuppessants (1 uM) for 48 hours (n = 6).

E, TNF-α protein level was quantified by ELISA in M0 macrophages after HEV infection and treatment by immunosuppessants (1 uM) for 48 hours (n = 4).

F, Cell viability of macrophages after HEV infection and treatment by immunosuppessants for 48 hours (n = 6).

Supplementary Figure 2

Supplementary Fig. 2

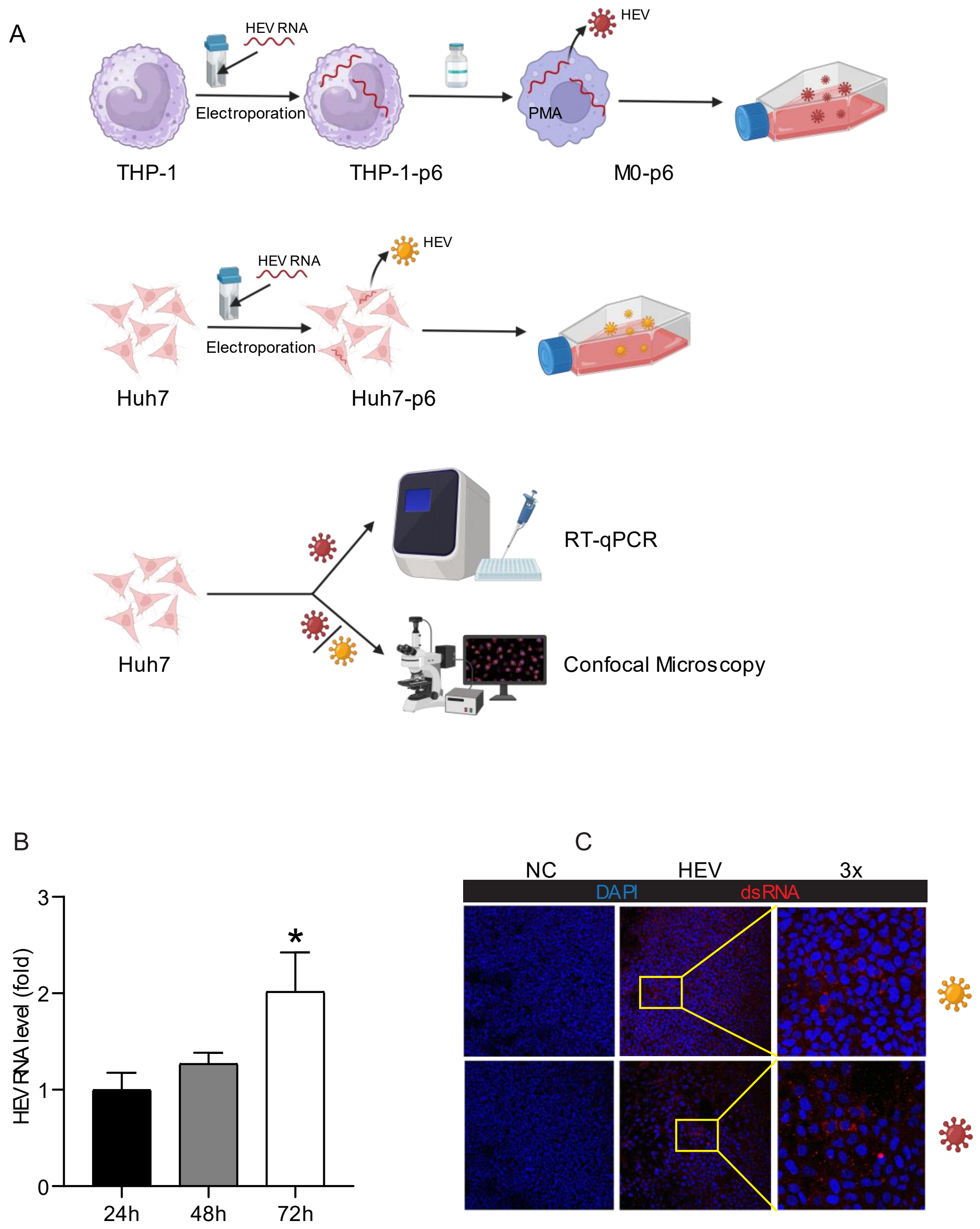

A, Schematic overview of harvesting HEV and characterizing inoculated Huh7 cells. The red and yellow HEV depict viral particles obtained from M0-p6 cells via secretion and from Huh7-p6 cells (as positive control) via the classical freeze-thaw method, respectively. The schematic illustration was generated in Biorender.
B, Quantitative RT-qPCR analysis of HEV viral level in Huh7 cells inoculated with HEV from M0-p6 cells via secretion, and cultured for 24, 48 or 72 hours (n = 6). *$P<0.05$, Mann-Whitney test, compared with the 24 hour time point.
C, Huh7 cells were inoculated with HEV for 72 hours. Viral dsRNA (red) and nucleus marker DAPI (blue) were examined under confocal microscopy.
